# Tracking inflation on a daily basis

**DOI:** 10.1186/s41937-020-00062-w

**Published:** 2020-11-04

**Authors:** Santiago E. Alvarez, Sarah M. Lein

**Affiliations:** 1 University of Basel, Basel, Switzerland; 2 CEPR, London, UK; 3 KOF ETH Zurich, Zurich, Switzerland

**Keywords:** Daily price index, Scraped online price data, Debit card expenditures, Real-time information

## Abstract

Using online data for prices and real-time debit card transaction data on changes in expenditures for Switzerland allows us to track inflation on a daily basis. While the daily price index fluctuates around the official price index in normal times, it drops immediately after the lockdown related to the COVID19 pandemic. Official statistics reflect this drop only with a lag, specifically because data collection takes time and is impeded by lockdown conditions. Such daily real-time information can be useful to gauge the relative importance of demand and supply shocks and thus inform policymakers who need to determine appropriate policy measures.

## Introduction

The COVID19 pandemic has led to dramatic changes in expenditures across product categories. Moreover, prices may reflect both negative demand and supply shocks, which have arguably affected the economy to an unprecedented degree. This makes it difficult for statistical agencies to accurately measure consumer prices in real time because expenditures are usually collected at a low frequency, and price collection is partially impossible because the retail outlets where statistical agencies usually collect prices are closed.

Additionally, policymakers must counter the crisis with the appropriate measures. These may differ depending on the relative importance of supply and demand shocks. The large decline in overall aggregate production or nominal consumption cannot inform on this because negative demand and supply shocks move quantities in the same direction. Furthermore, prices reflect these opposing forces since demand and supply shocks of the same sign push prices in opposite directions. This makes a daily price index a useful source of information for

____________________

^1^See, for example, Brown et al. (2020), Carvalho et al. (2020), Baker et al. (2020), Coibion et al. (2020), or Andersen et al. (2020).

^2^See Diewert and Fox (2020) for a detailed exposition of the problems surrounding CPI construction and data collection during the pandemic. policymakers.

In this paper, we construct a daily price index based on scraped online price data and expenditure weights based on debit card transactions by product category for Switzerland. This index allows us to monitor changes in the price level in real time and on a daily basis. We complement this index with data on the consumer price index (CPI) for categories for which we lack online prices or high-frequency changes in expenditure weights. We first show that the index is close to the official CPI before the lockdown, suggesting that we measure the same underlying dynamics. We then show that prices declined immediately after the lockdown, information that becomes available in official CPI figures only much later. Compared to the week before the lockdown, the daily price index declines by approximately 0.4% immediately after the lockdown and by approximately 0.7% until the time of this writing (the second week of July 2020). Using online prices during the lockdown can also be useful because many purchases have to be made online since retail stores are closed (for example, purchases of apparel). According to recent evidence based on point-of-sale transaction data, online retail payments related to e-commerce more than doubled during the lockdown period, compared to the same period in 2019 (Kraenzlin et al. 2020)^1^. Thus, with local retail stores being closed, online prices arguably reflect most of the purchases made during that period.

We show two applications for which such high-frequency data could be informative. First, we can observe both changes in quantities and prices by sector from before the lockdown to the period where many businesses were closed. Changes in prices and expenditures are very heterogeneous across sectors. We show that expenditures on food and beverages (at home) increase somewhat in total, and also prices increase. Meanwhile, prices and expenditures in categories are directly (accommodation and restaurants, entertainment, personal and professional services, other retail) and indirectly (transport) affected by the lockdown decline. Observing prices and quantities moving in the same direction suggests that, while clearly supply and demand shocks are both present, demand shocks are somewhat more prevalent at the moment, suggesting a slightly positive demand shock in the food at home category, and negative ones in the other categories named above. Using a daily price index by category allows us to monitor these sectoral developments closely, since the strength of demand and supply shocks may fade more or less quickly.

Second, we can ask whether prices are more or less flexible during and after the lockdown period?^2^ Looking at weekly frequencies of price adjustments, we do not find a significant increase or decline in the frequency of price adjustments during the lockdown period. However, when looking at the different product categories, we find a somewhat higher frequency of price increases in the food and beverages category, while price adjustment frequencies in the other sectors are either stable or decline slightly. Here, too, monitoring the frequency of price adjustment on a high-frequency and real-time basis may turn out useful in the aftermath of the lockdowns to track potential inflationary or deflationary pressures.

This paper is related to Diewert and Fox (2020), who suggest using online prices and real-time expenditure weights to construct the CPI during lockdown conditions. Our paper is an attempt to create such an index. It is also related to the literature on scraped online price data and their use in measuring the cost of living. Cavallo (2017) shows that online prices are similar to offline prices, suggesting that at least some of the prices underlying CPI calculations could be collected using scraping tools^3^. We show that replacing approximately 25% of the CPI basket with online prices results in very similar dynamics to those of the official monthly CPI before the COVID19-related lockdown. Our paper is therefore also related to the recent studies that monitor the economic consequences of COVID19, in particular the effects on inflation^4^. Balleer et al. (2020) use a monthly business tendency survey from Germany to infer the response of the price level to the COVID19 shock using firms’ responses to questions about their prices in the coming months. They find that prices tend to decline, consistent with what our index shows for Switzerland^5^.

Our work also relates to Cavallo (2020) and Seiler (2020). They show that updating the weights of the official CPI with changes in credit or debit card expenditures by product category results in higher aggregate price levels after lockdowns than those reported in official CPI figures. This is because consumers tend to switch expenditures towards product categories with relatively higher inflation rates (mostly food and beverages). Consistent with their findings, our price level is also higher when using CPI prices and debit-card expenditure adjusted weights and prices from the official CPI. Our main contribution in this paper is that we also use online prices, in addition to these adjusted expenditure weights. This allows us to track, in addition to changes in expenditure weights, also daily changes in prices. Because online prices during the lockdown declined somewhat more than official CPI prices, we observe overall a decline in the aggregate price level in our index.

Furthermore, our results on sectoral heterogeneity in responses of prices and quantities are related to Baqaee and Farhi (2020) and Guerrieri et al. (2020). Both show that differences across sectors are important to understand the propagation of (sectoral) supply and demand shocks. Monitoring both changes in quantities and prices for different product categories (or sectors) can thus be informative for the debate over whether the COVID19 shock is more of a supply or demand shock (see, for example, Baldwin and Weder di Mauro (2020), Balleer et al. (2020), and Brinca et al. (2020)).

This paper is structured as follows. In Section 2, we describe the online price data and the construction of price indexes. In Section 3, we report the price indexes up to the most recent data point as of this writing. We also discuss potential biases in official statistics during the lockdown. Section 4 documents the frequency of price adjustments in the aggregate and by category. Section 5 draws some conclusions.

## Data and methodology

Data for prices have been scraped from various websites on a daily basis since May 2018 for supermarket goods and since May 2019 for other categories, such as clothing, electronics, furniture, and heating oil. See Alvarez (2020) for a more detailed description. In this study, we focus on the data starting in May 2019 because we have a broader set of goods in the database. The data were extracted from six online retailers selling in the categories “Food, alcohol, and tobacco,” “Clothing and footwear,” “Heating oil,” “Furniture,” “Electronics,” “Office material,” and other supermarket items^6^. The majority of these retailers also have physical stores across Switzerland. These data allow us to identify products uniquely over time using shop-specific identifiers.

Table 1 provides an overview of the data and compares it to the official Swiss Federal Statistical Office (SFSO) main categories. Some of the categories are covered entirely by online prices such as “Food and non-alcoholic beverages” or “Clothing and footwear.” For some categories, such as “Housing and energy,” the substitution of official (SFSO) prices can be performed at lower levels of the CPI. Thus, online prices do not cover the entire main category weight (see Table 3 in Appendix 1 for a detailed overview of the replaced categories at different levels of aggregation). As services account for approximately 60% of the CPI basket weight, we are able to update the index with daily online data representing more than half of the weight for goods. The total number of products used for this analysis is 75,311^7^.

**Table 1:** Used CPI basket and weights

	Source		Weight			
Name	Prices	Weight	SFSO	Online	Lockdown	Prod.
Food and non-alcoholic beverages	Online	Debit card	10.54	10.54	14.93	8221
Alcoholic beverages	Online	Debit card	2.76	2.76	3.91	351
Clothing and footwear	Online	Debit card	3.4	3.4	.91	26,223
Housing and energy	Online*	SFSO	24.96	.69	33.14	9
Household goods and services	Online*	SFSO	3.79	3.35	5.03	13,679
Healthcare	Online*	SFSO	15.69	.21	20.83	47
Transport	SFSO	Debit card	10.97	0	8.08	0
Communications	Online*	SFSO	2.94	.17	3.91	691
Recreation and culture	Online*	Debit card	8.37	2.12	4.509	22,778
Education	SFSO	SFSO	1	0	1.32	0
Restaurants and hotels	SFSO	Debit card	9.46	0	1.17	0
Other goods and services	Online*	Debit card	6.12	1.59	1.92	3312
Total	Online*	Debit card*	100	24.502	100	75,311

Categories in which source contains an asterisk symbol (*) are categories in which part of their weight was substituted either with online data or with debit card data, but at lower levels of the CPI basket (see Table 3 in Appendix 1 for the exact matching). SFSO weights are the official CPI basket weights, online weights indicate the part out of the official weights covered by online prices, and lockdown weights are weights for the first week after the lockdown adjusted using credit card transaction data

**Fig. 1:**
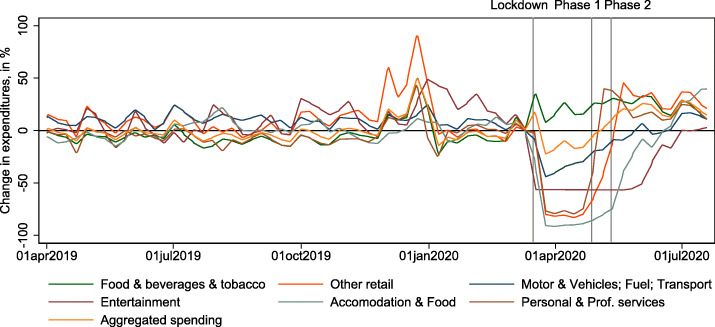
Changes in expenditures by category. This figure shows the 7-day moving averages of weekly deviations of total expenditures by category, relative to the week before March 16, 2020, the date of the lockdown in Switzerland. The vertical lines indicate the dates of the lockdown (March 16, 2020) and the phases of reopening (April 27, 2020, and May 11, 2020). Aggregated spending is the sum of all components shown here. Data source: http://monitoringconsumption.org/switzerland

To construct representative consumption baskets, we use the product category weights provided by the SFSO. Beginning in January 2020, we update these weights to reflect changes in consumption patterns before, during, and after the lockdown, as suggested in Cavallo (2020) and applied for the Swiss CPI in Seiler (2020). Daily real-time data for quantities per product category are taken from daily debit card expenditures published by the Monitoring Consumption Initiative for Switzerland^8^. We sum expenditures by category and week over regions (Grossregion). We sum the three categories “Motor and Vehicles,” “Fuel,” and “Transport,” because they are all included in the CPI category (“Transport”). We use weekly data because the daily data are noisier due to day-of-the-week effects (very small numbers of transactions on Sundays). We show the expenditure data by category relative to the week before the first lockdown phase that began on March 16, 2020, in Fig. 1,^9^.

These shifts in consumption expenditures are then reflected in changes in CPI category weights during the lockdown. For example, the weight of the category “Food and non-alcoholic beverages” increases by almost 50% from 10.5 to 15.5% (Table 1, comparing the third with the fifth column). Meanwhile, the weight of “Restaurants and hotels” declines from 9.5 to only 1.2%. Related to these expenditure shifts, relative expenditures on categories, where nominal expenditures remain mostly constant, go up. “Housing and energy,” for example, includes rents (a weight of 24.3% in the total CPI), which probably do not change much during the lockdown. Since aggregate expenditures on the debit card categories decline by up to 25% (Fig. 1), the relative weight on rents increases to 35%. Rents are arguably not paid with debit cards, but via regular bank account transactions^10^.

One caveat of the debit card expenditure data is that it includes only debit cards and not credit cards. Arguably, online spending is mostly done via credit card transactions. This online spending is thus probably not included in our weights and may overstate the decline in retail products that were not available in closed stores, but still available online^11^. Our main price index, as we describe below, is an average of an index that fully reflects these expenditure shifts (Paasche) and an index that does not reflect these shifts (Laspeyres). This potential overstated decline is therefore muted in our main price index (Fisher).

To compute the price index on a daily basis, we proceed in two steps. First, we use the CPI weights, which do not reflect changes in consumption due to the lockdown. We replace prices in the CPI with daily online prices for all categories with online prices, as shown in Table 1. For each category *j*=1...*J*, we construct a category-level Jevons index over the set of *i*=1...*N* products observed in the base period, which is the week before the lockdown (9 March 2020 to 15 March 2020) as:

1 $$ P_{j}^{t} = \prod_{i=1}^{N} \left(\frac{P_{i}^{t}}{P_{i}^{0}}\right)^{\frac{1}{n}}.  $$

We construct a daily version of a Laspeyres (1871) price index:

2 $$ P_{Las}^{t} = \sum_{i=1}^{J} \frac{P_{j}^{t}}{P_{J}^{0}} w_{j}^{0,CPI}\\  $$

where $P_{j}^{t}$ equals the price index for online goods in Eq. 1 or the CPI category price index from the SFSO where online prices are not available. The weight $w_{j}^{CPI}$ is from the CPI and thus does not reflect contemporaneous changes in consumption patterns due to the pandemic.

We then construct a daily version of a Paasche (1874) price index:

3 $$ P_{Paa}^{t} = \left[\sum_{i=1}^{J} \left(\frac{P_{j}^{t}}{P_{J}^{0}}\right)^{-1} w_{j}^{t,COVID19}\right]^{-1}  $$

where we include the COVID19-adjusted current-day weights and measure the price of the COVID19 basket at prices in the base period.

As is well known, the Laspeyres (Paasche) index tends to be upward (downward) biased in normal periods because consumers substitute towards products that become relatively cheaper. This means that the Laspeyres index tends to underweight the products that become cheaper, while the Paasche index overweights them. However, during the lockdown period, consumers substantially shift expenditures towards food at home and away from categories that are produced by sectors that are temporarily shut down. This substitution is not a result of relative price shifts but of many products not being available.

The Fisher index, calculated as the geometric average of the Paasche and Laspeyres indexes, should be unbiased in normal periods because it averages out the upward and downward biases of the Laspeyres and Paasche indexes, respectively. The index is thus:

4 $$ P_{t}^{Fis} = \left(P_{t}^{Paa} \cdot P_{t}^{Las}\right)^{0.5},  $$

which we use as our main index reflecting changes in both expenditures and prices.

## Daily price indexes before, during, and after the lockdown

This section first shows how the daily Fisher price index compares to the official monthly CPI when considering a longer horizon. It then shows the lockdown period in particular and discusses biases arising from large shifts in consumption patterns.

**Fig. 2:**
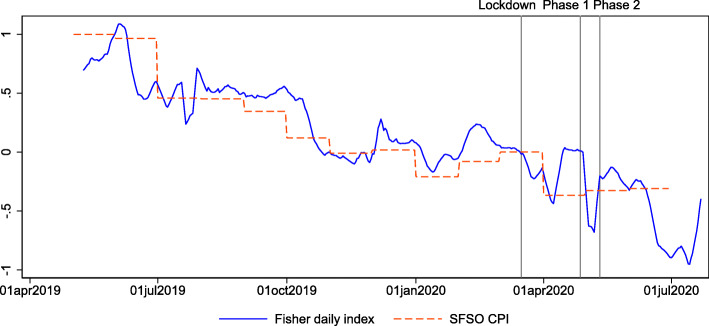
Daily price indexes from May 2019 to July 2020. This figure shows the Fisher price index based on daily online prices and daily credit card expenditures (blue solid line; 7-day lagged moving average) and the official monthly CPI (red dashed line). The vertical lines indicate the dates of the lockdown (March 16, 2020) and the two phases of reopening (April 27, 2020, and May 11, 2020). The figure spans the period May 1, 2019, to July 23, 2020

Can online prices track official statistics at all? Figure 2 plots the 7-day moving average of the daily price index (in logs) together with the official CPI statistics since mid-2019. The longer history of this daily price index shows that it fluctuates around the official index in 2019, even though it includes only online prices for approximately 25% of the total sample. This is consistent with the results in Cavallo (2017) that online and offline prices are similar in normal times and that online prices can be used as inputs for CPI calculations instead of offline prices. While Fig. 2 includes the CPI prices for categories, for which we do not have online prices, the similarity is not only driven by these categories. Figure 7 in Appendix 1 shows the comparison of online prices with those of the CPI only for the categories where we could replace CPI prices with online prices. The dynamics are similar.

**Fig. 3:**
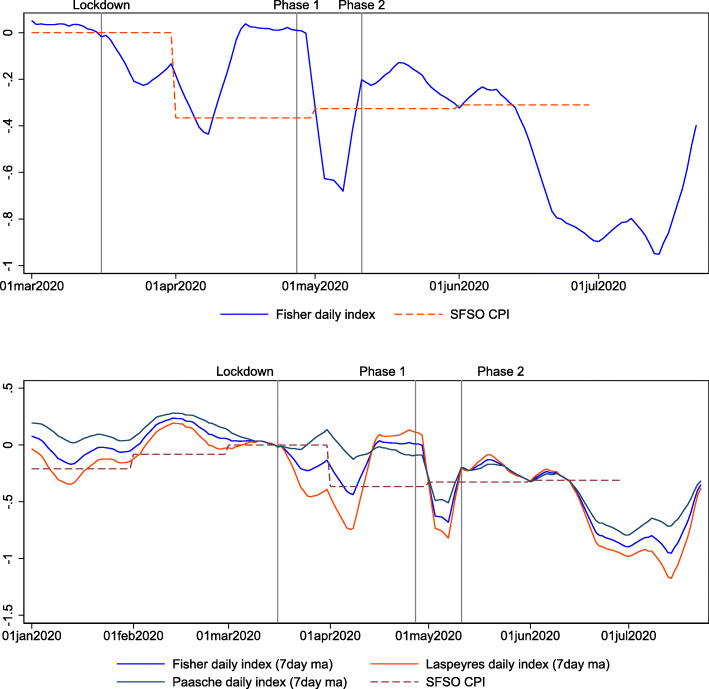
Daily price indexes in 2020. The upper panel in this figure shows the Fisher price index based on daily online prices and daily credit card expenditures (blue solid line; 7-day lagged moving average) and the official monthly CPI (red dashed line) around the lockdown and reopening period. The lower panel shows the Fisher (blue), Laspeyres (red), and Paasche (gray) indexes during the lockdown and reopening periods together with the official monthly CPI (red dashed line). The vertical lines indicate the dates of the lockdown (March 16, 2020) and the two phases of reopening (April 27, 2020, and May 11, 2020). The figure spans the period May 1, 2019, to July 23, 2020

Figure 3 shows daily price indexes in 2020. The beginning of the lockdown is shown as a vertical line on March 16, and the beginning of the two reopening phases is shown for April 27 and May 11 (see also footnote 11). In the upper panel, we show the Fisher daily index and the official CPI around the lockdown and the reopening phases. The Fisher index shows that immediately after the lockdown, prices declined by approximately 0.4%. This information is available approximately 6 weeks earlier than the official index, which is released in early April for data collected for the month of March. The online index declines by a similar amount as the official index, after it has been updated with the prices that could be collected at the time^12^. At the time of writing, the price index is around 0.5% below the pre-lockdown level^13^. This suggests that, in the very short run, negative demand shocks dominate negative supply shocks, consistent with findings for Germany based on producer surveys (Balleer et al. 2020).

The bottom panel of Fig. 3 shows the three daily price indexes: Paasche, Laspeyres, and Fisher. The difference between the Laspeyres and Fisher indexes illustrates the extent of substitution bias. It is larger in the period after the lockdown, which reflects the large shifts in spending patterns depicted in Fig. 3. The bias amounts to up to 0.3 percentage points, which is approximately three times larger than the substitution bias estimated before the pandemic^14^. In normal times, the Laspeyres index tends to overestimate inflation because consumers substitute towards products that become relatively cheaper. In this case, we observe the opposite: consumers substitute towards product categories where prices were more or less stable (mostly food, beverages, and tobacco), while expenditures on product categories with falling prices decrease substantially. This is also reflected in the Paasche index, which is nearly stable (see Fig. 3, lower panel). This suggests that consumers substitute away from product categories that become relatively cheaper. This is because consumers cannot demand many of the goods from these categories due to lockdown restrictions or because tastes shift away from these goods. However, the bias is relatively short lived and becomes small again after the end of the lockdowns.

Shifts in prices and expenditures can also be compared across product categories, as it is very likely that some were affected more severely by demand shocks, while others were affected more by supply shocks (Baqaee and Farhi 2020). In general, prices and quantities tend to move in the same direction in the case of demand shocks, while they move in opposite directions in the case of supply shocks. Observing both changes in quantities and prices is thus interesting regarding the debate over whether the COVID19 shock is more of a supply or demand shock and how that differs across sectors.

**Fig. 4:**
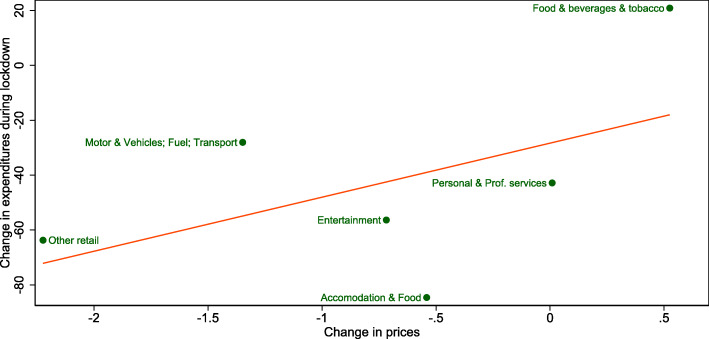
Relationship between the change in prices and change in expenditures during the lockdown. This figure shows a scatter plot of the change in average expenditures and average change in prices during the lockdown period from March 16, 2020, to May 11, 2020

Figure 4 plots the changes in prices and associated changes in spending. It shows that the price decline was particularly strong in the retail sector (excluding “Food, beverages, and tobacco”), which also shows a relatively large decline in expenditures (approx. −50*%*). Similar movements, albeit less pronounced, can be seen in the sector “Transport.” These falling prices and even greater reductions in expenditure are typically accompanied by a negative demand shock. Consumer spending falls most sharply in the “Hotels and restaurants” and “Leisure and culture” sectors, which were not allowed to open or only partially open. Here, too, prices fall slightly, albeit less sharply than in the sectors mentioned above. Expenditures also fall in the “Services” sector, with prices remaining almost unchanged. This would indicate that here, the demand and supply shocks are roughly balanced. In the “Food, beverages, and tobacco” sector, which was not affected by the lockdown, spending actually increased while prices remained stable. This would indicate an approximately balanced expansion of demand and supply in this sector. This is consistent with anecdotal evidence that, although initial demand in supermarkets soared just before and after the lockdown due to stockpiling motives, supply was generally not constrained^15^.

## Price setting behavior before, during, and after the lockdown

**Fig. 5:**
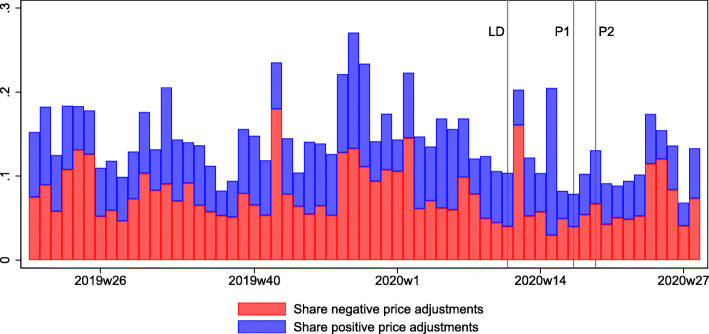
Share of price adjustments. This figure shows the fraction of price increases and decreases (as a share of all prices observed) on a weekly basis (that is, a price change is observed if a price changes from 1 week to the next). Red bars are price decreases, and blue bars price increases. The bars are stacked, such that the total length of the bar indicates the total fraction of price changes per week. LD, P1, and P2, stand for lockdown, phase 1, and phase 2, respectively

How flexibly do prices respond to the lockdown? For answering this question, we first show the share of all included products that adjust their prices on a weekly basis (see Fig. 5, which plots the frequency of positive and negative price changes in stacked bars). There is no significant change in the frequency of price adjustments when looking at all categories together. This, however, might be caused by different changes on pricing behavior by categories of goods. Furthermore, there is no clear change in the frequency of positive or negative price changes.

**Fig. 6:**
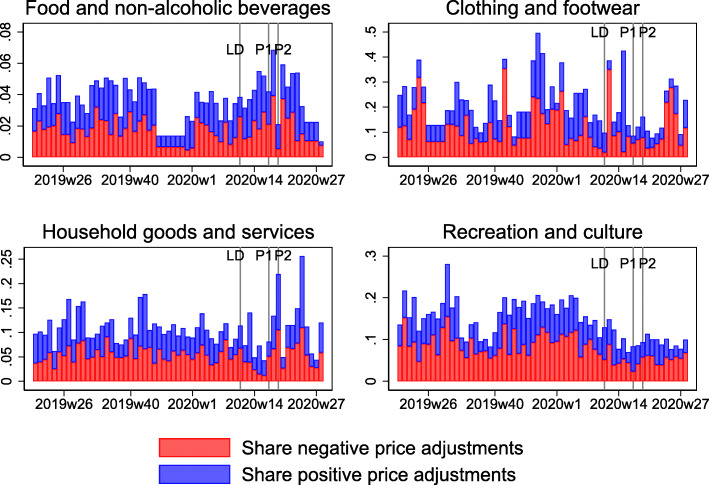
Share of price adjustments by category. This figure shows the fraction of price increases and decreases (as a share of all prices observed) on a weekly basis (that is, a price change is observed if a price changes from 1 week to the next) for selected product categories. Red bars are price decreases, and blue bars price increases. The bars are stacked, such that the total length of the bar indicates the total fraction of price changes per week. LD, P1, and P2, stand for lockdown, phase 1, and phase 2, respectively

Table 2 shows the average share of price adjustments across the weeks included in each time interval for the four categories “Food and non-alcoholic beverages,” “Clothing and footwear,” “Household goods and services,” and “Recreation and culture.” Similar to the heterogeneity in price and expenditure changes across categories reported above, there are some differences across categories in the frequency of price adjustments. While price adjustments in “Food and non-alcoholic beverages” become somewhat more prevalent during the two phases of the lockdown (first row in Table 2 and upper left panel in Fig. 6), the price adjustments in the category “Recreation and culture” become less frequent (fourth row in Table 2 and lower right panel in Fig. 6). Prices change less frequently during the lockdown in the category “Household goods and services,” but more frequently after the lockdown, and with more positive price adjustments (third row in Table 2 and lower left panel in Fig. 6). The frequency of price adjustment in the category “Clothing and footwear” is somewhat lower on average (second row in Table 2 and upper right panel in Fig. 6) between phases 1 and 2, but it is very volatile overall with weeks that show up to 50% of all prices changing (the scales across categories differ in Fig. 6). This is likely due to frequent sales in this category^16^.

**Table 2:** Averages of weekly shares of price adjustments by period

	Period			
Category	Before LD	LD–P1	P1–P2	After P2
Food and non-alcoholic beverages	.0354	.0429	.0553	.0344
Clothing and footwear	.2181	.2268	.104	.1745
Household goods and services	.106	.0823	.1077	.1238
Recreation and culture	.1555	.1087	.0851	.0939
All products	.1412	.1128	.0907	.116

Notes: This table shows the average share of price adjustments by product category and in total during all weeks by period. LD, P1, P2, stand for lockdown (3/16/2020), phase 1 (4/27/2020) and phase 2 (5/11/2020), respectively. For example, in the product category food and non-alcoholic beverages, the weekly share of price changes is computed for each week and then we measure the average of all weeks before the LD and report it in the first column. Total includes all observed products, not only the products of the four categories displayed

Sizes of price adjustments are similar before, during, and after the lockdown, as reported in Appendix 2.

## Conclusion

In this note, we propose a daily price index composed of daily scraped online prices for different product categories and debit card expenditures by product category. We update prices and weights of CPI categories for which we have this additional high-frequency information.

We show that the index reflects the official monthly CPI quite well in the period before the lockdown, thus confirming that online prices carry similar information as the prices that are included in the CPI. The index shows that prices decline immediately after the lockdown and remain approximately 0.4% lower than those in the week just before the lockdown was implemented, supporting recent evidence suggesting that negative demand shocks are somewhat larger than negative supply shocks. This is also the case for most product categories, where prices and expenditures both fell and thus suggest that demand shocks dominated at this point in time.

While our index can be useful for policymakers to track inflation in real time, we do not make any statements about the longer-term effects of the pandemic recession on inflation. However, since prices that consumers observe in their daily lives are an important ingredient of consumers’ inflation expectation formation process (D’Acunto et al. 2019), the daily inflation figures may carry some information about longer-term inflation expectations, which will be an important factor in determining inflation in the medium run.

## Appendix 1. Product categories with online prices

**Table 3:** Matched CPI categories

Level 2 ID	ID	Name	Level	Weight	Products
1	1002	Bread, flour, and cereal products	4	1.6	1554
1	1074	Meat, cold cuts, and sausages	4	2.28	701
1	1179	Fish and seafood	4	.37	257
1	1198	Milk, cheese, and eggs	4	1.6	1155
1	1284	Fats and edible oils	4	.26	143
1	1305	Fruit, vegetables, potatoes, and mushrooms	4	2.12	412
1	1448	Sugar, jam, honey/other sugary foods	4	.66	1223
1	1481	Other food products	4	.72	1828
1	1518	Coffee, tea, cocoa, and nutritional beverages	4	.42	463
1	1544	Mineral waters, soft drinks, and juices	4	.51	485
2	2	Alcoholic beverages and tobacco	2	2.76	351
3	3	Clothing and footwear	2	3.4	26,223
4	4090	Heating oil	4	.69	9
5	5001	Furniture, furnishings, and floor coverings	3	1.36	5465
5	5070	Household textiles	3	.3	241
5	5100	Household appliances	3	.57	6299
5	5140	Glassware, tableware, and household utensils	3	.29	280
5	5200	Tools for house and garden	4	.33	106
5	5221	Goods for routine household maintenance	4	.5	1288
6	6070	Medical products	4	.21	47
8	8006	Telecommunication equipment	3	.18	691
9	9001	Audiovisual, photographic, and IT equipment	3	.79	9182
9	9211	Games, toys, and hobbies	4	.37	12,713
9	9300	Plants, flowers, and garden products	4	.48	289
9	9555	Writing and drawing materials	4	.14	594
12	12021	Personal hygiene articles	4	.93	2741
12	12150	Electrical appliances for personal care	4	.05	421
12	12160	Personal effects	3	.61	150
Total	–	–	–	24.502	75,311

Weights as in the official CPI for 2020

**Fig. 7:**
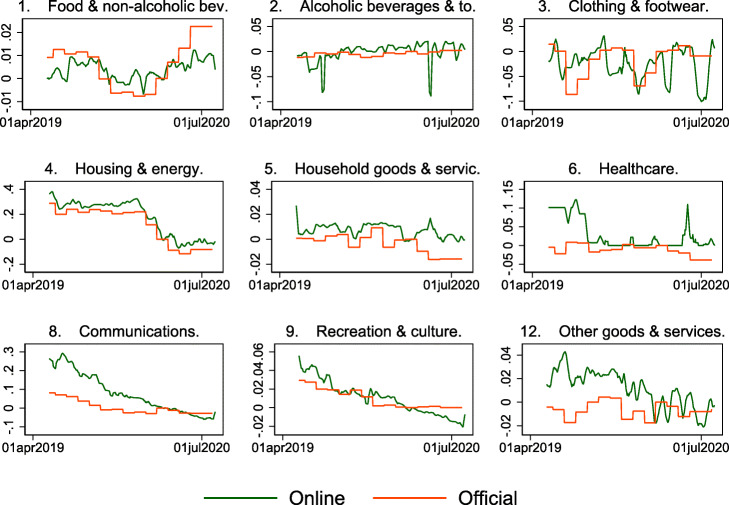
Only matched positions aggregated at SFSO category level 2. This figure shows the official and online inflations aggregated at SFSO category level 2 keeping only the lower-level positions available online. Constant official weights for 2020 used

## Appendix 2. Size of price adjustments

**Fig. 8:**
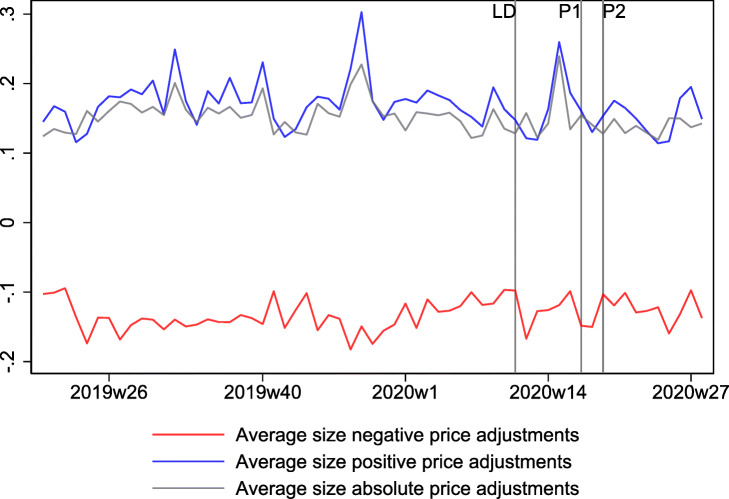
Size of price adjustments. This figure shows the average nonzero size of price adjustments. LD, P1, and P2, stand for lockdown, phase 1, and phase 2, respectively

**Fig. 9:**
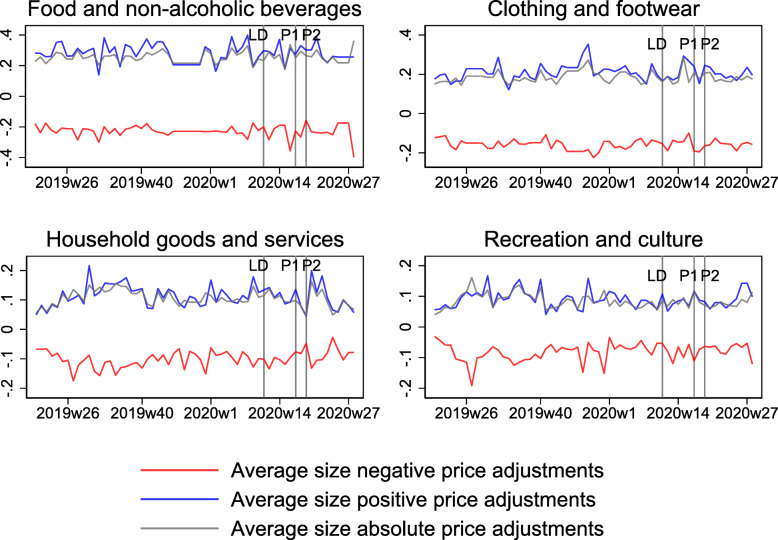
Size of price adjustments by category. This figure shows the average nonzero size of price adjustments by product category. LD, P1, and P2 stand for lockdown, phase 1, and phase 2, respectively

## Appendix 3. Changes in expenditures by transaction type

**Fig. 10:**
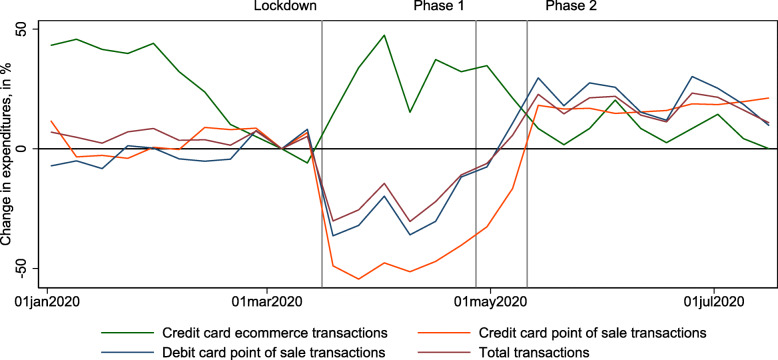
Changes in expenditures by transaction type. This figure shows weekly deviations of total expenditures by transaction type, relative to the week before March 16, 2020, the date of the lockdown in Switzerland. The vertical lines indicate the dates of the lockdown (March 16, 2020) and the phases of reopening (April 27, 2020, and May 11, 2020). Data source: http://monitoringconsumption.org/switzerland

## Data Availability

We have posted the daily inflation data on our website. It is thus publicly available.

## Abbreviations

CEPR: Centre for Economic Policy Research
COVID19: Coronavirus (19)
CPI: Consumer price index
KOF: KOF Swiss Economic Institute
LD: Lockdown (March 16, 2020)
P1: Reopening phase 1 (April 27, 2020)
P2: Reopening phase 2 (May 11, 2020)
SFSO: Swiss Federal Statistical Office

## Footnotes

1 The shift towards e-commerce is also shown in the monitoring consumption data in credit card expenditures shown in Fig. 10 in Appendix 3.

2 This question is related to the empirical literature on state- versus time-dependent pricing. One of the main findings in this literature is that the frequency of price adjustment looks very stable in periods where aggregate shocks are not very large on average, but the frequency of price adjustment can vary a lot when shocks are large, as shown for example in Gagnon (2009), Karadi and Reiff (2010), and Auer et al. (2018).

3 See also Cavallo and Rigobon (2016) and Cavallo et al. (2018).

4 For daily indicators of economic activity, see, for example, Burri and Kaufmann (2020), Eckert and Mikosch (2020), Eichenauer et al. (2020), or Lengwiler (2020).

5 Overall, recent research suggests that price responses differ across sectors. Burghof et al. (2020) collect online prices for five supermarkets in Germany. They find a slight increase in these prices of approximately 0.8% between February and April.

6 These retailers are Interdiscount, Mediamarkt, Coop, Ikea, Zalando, and Heizoel.ch. We selected these retailers because the format of their website was suited for scraping and they are representative online and offline stores for several components of the CPI. These stores tend to be large retailers, but of course they represent only a share of all online purchases. For example, rather upscale stores are not included. We plan to add more such stores in the future.

7 Note that these can be a given good but identified at two different retailers (e.g., one specific PC that is sold by two different retailers) or in the case of heating oil the same good sold across different locations.

8 See http://monitoringconsumption.org/switzerland, which is a joint initiative of the University of St. Gallen, the University of Lausanne, and Novalytica. The data are publicly available. They contain similar information to, for example, the OpportunityInsights data for the USA described in Chetty et al. (2020) and used in Cavallo (2020).

9 Switzerland had strict restrictions in place from March 16 to April 26 and opened lower-risk businesses and retail stepwise between April 27 and June 15, with openings of hairdressers, cosmetic studios, do-it-yourself stores, flower shops, and garden centers in the first step, and shops, restaurants, markets, museums, and libraries in the second step (as of May 11).

10 The decline of approximately 25% is also consistent with aggregate debit and credit card data shown in Fig. 10 in Appendix 3. Since credit card transactions are not available as a breakdown into merchant categories, we cannot use them to infer the expenditure weights, but the total spending pattern is consistent with the one based on debit cards only.

11 In Fig. 10, we show that total expenditures on credit cards and debit cards display similar dynamics, where online expenditures with credit cards seem to just offset the larger decline in credit card point-of-sale transactions (compared to debit card point-of-sale transactions), such that the total credit card and debit card spending shows similar dynamics as debit card spending alone.

12 According to press releases from the SFSO, approximately 20% of all prices could not be collected in April. This share increases to 25% for the sectors most affected by the pandemic.

13 The large swing in June is mostly due to the category “clothing,” where we had observed a large share of seasonal sales.

14 This is also consistent with Diewert et al. (2009), who report a substitution bias of 0.13% for the Swiss CPI for the period 1993–2002

15 One caveat in this analysis is that we use only prices that are available already before the lockdown to calculate price changes. An ideal price index that would measure the changes in cost of living from a welfare perspective would include a term adjusting for changes in variety, since some products may not be available during the lockdown, and possibly a term that adjusts for taste shifts during the lockdown. Such an index would be the one proposed by Redding and Weinstein (2019), for example, applied for the pre-lockdown period to Swiss data in Braun and Lein (2019). Since we do not observe product-specific expenditure shifts, it would be difficult to construct such an index without stronger assumptions about within-product category expenditure shifts. Furthermore, we would like to remain close to the official methodology to calculate prices, which is a Laspeyres-type index.

16 Also, products traded online have on average higher price adjustment frequencies as suggested in Rudolf and Seiler (2020), who look at Swiss microdata underlying the CPI.
